# Effect of Heading Date on the Starch Structure and Grain Yield of Rice Lines with Low Gelatinization Temperature

**DOI:** 10.3390/ijms231810783

**Published:** 2022-09-15

**Authors:** Naoko Crofts, Kaito Hareyama, Satoko Miura, Yuko Hosaka, Naoko F. Oitome, Naoko Fujita

**Affiliations:** Department of Biological Production, Akita Prefectural University, 241-438 Kaidobata-Nishi, Shimoshinjo-Nakano, Akita City 010-0195, Japan

**Keywords:** rice, heading date, starch, yield, low gelatinization temperature, starch synthase IIa

## Abstract

Early flowering trait is essential for rice cultivars grown at high latitude since delayed flowering leads to seed development at low temperature, which decreases yield. However, early flowering at high temperature promotes the formation of chalky seeds with low apparent amylose content and high starch gelatinization temperature, thus affecting grain quality. Deletion of starch synthase IIa (SSIIa) shows inverse effects of high temperature, and the *ss2a* mutant shows higher apparent amylose content and lower gelatinization temperature. *Heading date 1* (*Hd1*) is the major regulator of flowering time, and a nonfunctional *hd1* allele is required for early flowering. To understand the relationship among heading date, starch properties, and yield, we generated and characterized near-isogenic rice lines with *ss2a* *Hd1*, *ss2a* *Hd1 hd1*, and *ss2a* *hd1* genotypes. The *ss2a Hd1* line showed the highest plant biomass; however, its grain yield varied by year. The *ss2a* *Hd1 hd1* showed higher total grain weight than *ss2a* *hd1*. The *ss2a* *hd1* line produced the lowest number of premature seeds and showed higher gelatinization temperature and lower apparent amylose content than *ss2a* *Hd1.* These results highlight *Hd1* as the candidate gene for developing high-yielding rice cultivars with the desired starch structure.

## 1. Introduction

**Figure 1:**
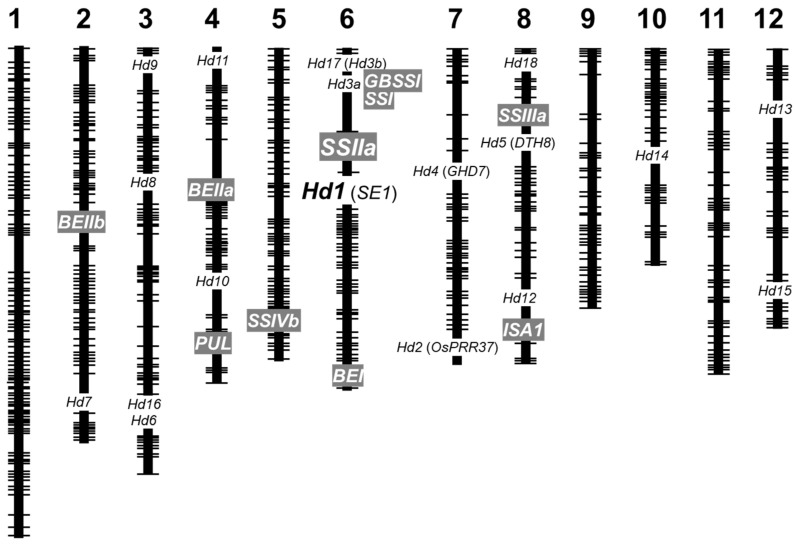
Chromosomal locations of genes responsible for the regulation of heading date and endosperm starch biosynthesis in rice. Genes controlling heading date are written in black writing (*Hd1* is enlarged) and those involved in starch biosynthesis in the rice endosperm are highlighted in gray. Note that *Hd1* and *SSIIa* are located in close proximity of each other on chromosome 6.

Heading date is one of the most important agricultural traits, particularly for rice (*Oryza sativa* L.) cultivars cultivated in high-latitude areas, because early flowering ensures seed development at optimum temperature during the short summer, thus maximizing yield [1]. Cultivars with different heading dates have been selected at different latitudes through natural and artificial means [1,2,3]. Several genes governing heading date have been identified in rice [4,5,6,7,8,9,10,11,12,13,14,15] and are shown in Figure 1. *Heading date 1* (*Hd1*) encodes a zinc-finger protein and is the major determinant of heading date [4]. *Hd1* represses the expression of florigen, *Hd3a*, under a long-day photoperiod but promotes its expression under short days [5,14,16]. Once heading is initiated, flowering generally occurs within a couple of days. Therefore, the nonfunctional *hd1* allele is required for early flowering under long-day conditions. Different rice cultivars have acquired several single nucleotide polymorphisms (SNPs) in *Hd1* during the process of domestication [2,3,17,18].

Starch, the major component of rice grain, is composed of glucose polymers of essentially linear amylose and precisely, but highly, branched amylopectin [19,20]. The ratio of amylose to amylopectin as well as the length and frequency of amylopectin branches affect the physicochemical properties of starch and transparency of grains, thus affecting the quality of rice [21,22,23]. The amylose found in rice endosperm is exclusively synthesized by granule-bound starch synthase I (GBSSI); thus, the expression level of *GBSSI* determines the amylose content of rice grains. Polymorphisms at the last nucleotide of the first intron of the *GBSSI* gene are commonly seen in japonica rice (*O. sativa* L. ssp. *japonica*) [24,25,26,27] and are known to reduce the splicing efficiency of *GBSSI* mRNA, especially under high temperature during seed development, which decreases GBSSI protein production and consequently amylose content [28,29,30].

Amylopectin is synthesized in the rice endosperm by the synergistic and balanced actions of multiple isozymes of starch synthases (SSs), branching enzymes (BEs), and debranching enzymes by forming multiprotein complexes [31,32]. Chromosomal locations of genes encoding these isozymes are summarized in Figure 1. According to the current understanding of amylopectin biosynthesis, SSIIIa synthesizes long glucan chains (amylopectin backbone) with degree of polymerization (DP) > 30, and BEI generates long branches. BEIIb generates short amylopectin branches with DP 6–7, and SSI elongates these short branches to DP 8–12. SSIIa further elongates these branches to DP 12–24 in most indica rice (*O. sativa* L. ssp. *indica*) cultivars, but the SSIIa isozyme of japonica rice is less active than that of indica rice and produces fewer intermediate chains with DP 12–24 [33]. Unnecessary branches are trimmed off by debranching enzymes such as isoamylase 1 [31,34]. Rice lines lacking BEIIb exhibit fewer short amylopectin chains, greater long amylopectin chains, and consequently higher gelatinization temperature than the wild type [35,36,37,38]. High temperature during seed development also impacts the expression level and activity of BEIIb [39,40,41,42], which increases the long amylopectin branch chains, gelatinization temperature, and chalky seed frequency and decreases the palatability of cooked rice [39,42].

The effects of SSIIa loss on amylopectin structure are opposite to those of BEIIb loss, although loss of either one of these enzymes has the same effect on amylose content. A *ss2a* null mutant rice line, EM204, was previously isolated from the N-nitroso-N-methylurea (NMU)-treated mutant panel of the japonica rice cultivar Kinmaze [43]. EM204 harbors a point mutation at the last nucleotide of the intron 5 of *SSIIa*, resulting in the loss of exon 6 and no detectable SSIIa activity in developing seeds [43]. Loss of SSIIa activity increased short amylopectin branches with DP < 11 and lowered the gelatinization temperature by 5 °C compared with the parental line (Kinmaze), although Kinmaze and other typical japonica rice cultivars exhibit lower SSIIa activity than typical indica rice varieties [33,43]. In addition, loss of SSIIa activity increased the apparent amylose content to 24%, which was considerably higher than that of Kinmaze (20%) [43]. Both Kinmaze and EM204 flower in early September in Akita, Japan (39.7° N, 140.1° E). Although the starch of EM204 shows great potential as an anti-retrogradation agent, the agricultural traits of this mutant line, such as heading date and yield, need further improvement since nighttime temperature sharply declines in September, which drastically reduces grain yield, depending on the harvest year. Thus, EM204 was backcrossed twice with a high-yielding elite rice cultivar, Akita 63 [44], which flowers in early August. Although more than half of the backcrossed lines flowered in early August, some flowered in September because the *SSIIa* and *Hd1* genes are located in close proximity to each other on chromosome 6 (Figure 1).

A previous study analyzed the effects of different *Hd1* alleles on agronomic traits and amylose content using multiple genetic backgrounds, such as glutinous rice, japonica rice, and indica rice. However, because these rice genotypes harbor different alleles of *SSIIa* and *GBSSI*, in addition to the genes responsible for plant biomass and yield components [18], the effects of *Hd1* alleles on starch properties could not be evaluated properly. Therefore, in this study, we used Kinmaze (the parental line of EM204) and Akita 63, both of which have *ss2a^L^*, to identify the allele(s) responsible for the differences in heading dates. In addition, to accurately evaluate the effects of different heading dates on starch properties and agricultural traits in the absence of SSIIa, we backcrossed EM204 (late-heading *ss2a* mutant) with Akita 63 (early-heading elite rice cultivar) and generated near-isogenic lines (NILs) with three different combinations: *ss2a ss2a Hd1 Hd1* (*ss2a Hd1*), *ss2a ss2a Hd1 hd1* (*ss2a Hd1 hd1*), and *ss2a ss2a hd1 hd1* (*ss2a hd1*). The effects of three *Hd1* genotypes on agricultural traits, apparent amylose content, amylopectin structure, and starch gelatinization temperature, in the absence of SSIIa, are discussed.

## 2. Results

### 2.1. Nucleotide Sequence of Hd1 in Kinmaze, Akita 63, and Akitakomachi

**Figure 2:**
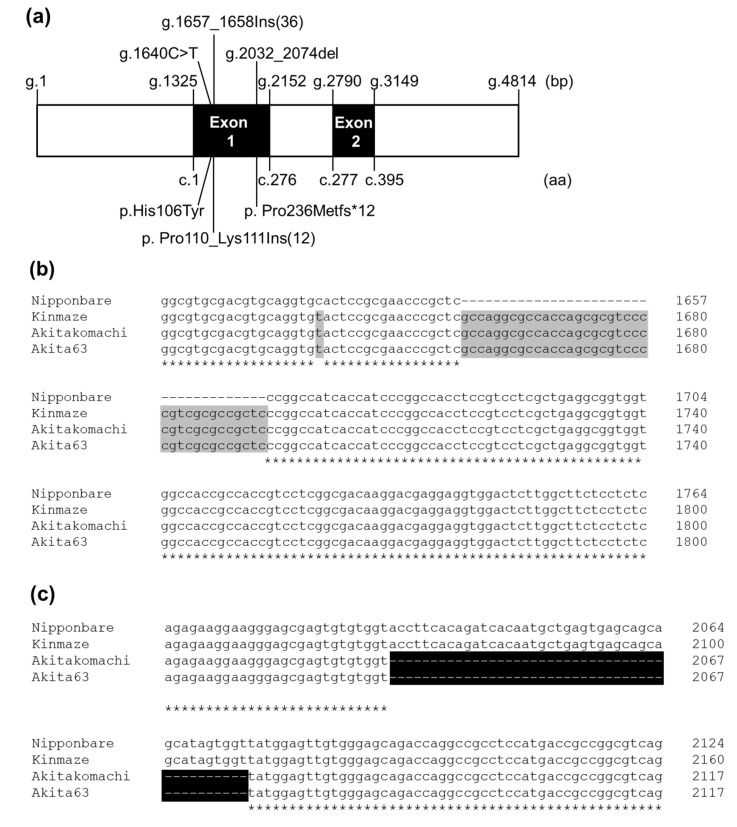

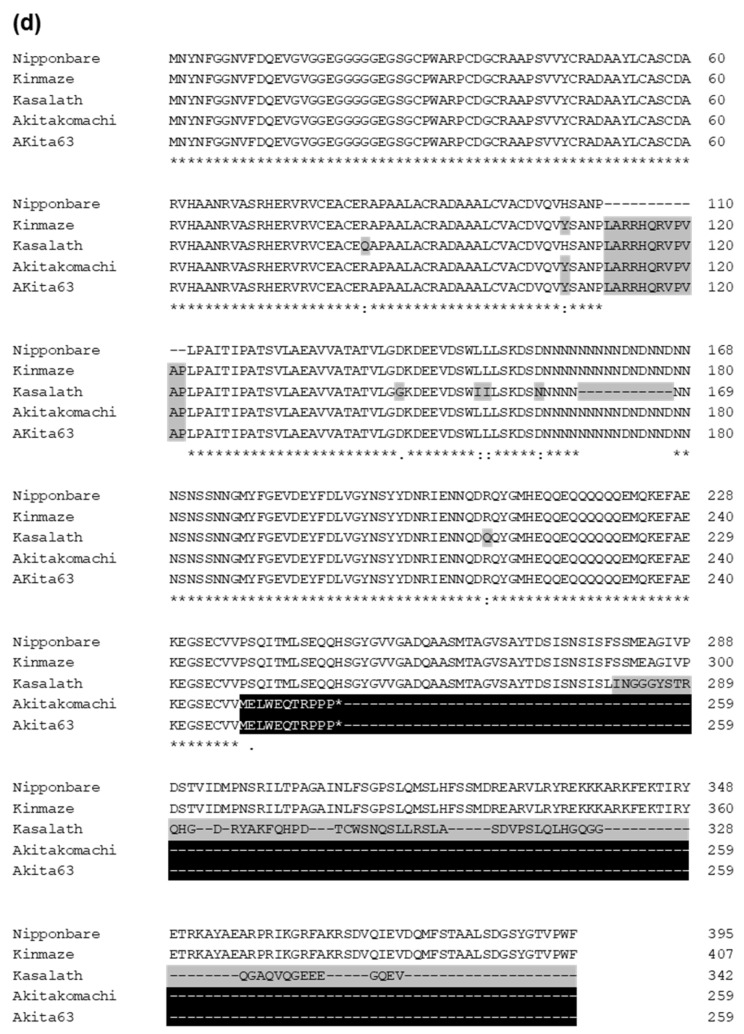
Comparisons of *Hd1* DNA sequences and deduced amino acid sequences in various rice lines. (**a**) Schematic representation of the *Hd1* gene structure in Akita 63. The positions of SNPs and resulting amino acid substitutions relative to Nipponbare are indicated. Ins, insertion; del, deletion; fs*12, frame shift-generated stop codon after 12 amino acids. The letter ‘g’ followed by a number indicates the nucleotide position in genomic DNA. Similarly, letters ‘c’ and ‘p’ followed by numbers represent the nucleotide position in cDNA and amino acid position in protein, respectively. Numbers in brackets indicate the number of nucleotide or amino acid insertions. (**b**,**c**) DNA sequence alignments of *Hd1* from 1598 to 1764 bp (**b**) and from 2005 to 2117 bp (**c**). The nucleotide positions correspond to the *Hd1* sequence of Nipponbare. (**d**) Full-length amino acid sequence alignment of Hd1. DNA and protein sequences different from Nipponbare are indicated with gray boxes, and regions missing in Akita 63 and Akitakomachi are indicated by black boxes. Sequences used to create the alignments are as follows: Nipponbare (AB041838), Kinmaze (MK449352), Kasalath (AB041839), Akitakomachi (MK449350), and Akita 63 (MK449351). Asterisks indicate identical nucleotides (**b**,**c**) and amino acid residues (**d**).

The *SSIIa* and *Hd1* genotypes and heading dates of different rice accessions are summarized in Table 1. Genomic DNA sequence of *Hd1* was amplified from Kinmaze, Akita 63, Akitakomachi, and Nipponbare using primers #5 and #12 (Table S1 and Figure S1) and compared (Figure 2b–d). Kinmaze is the parental line of EM204, which flowers in early September; Akita 63 is the high-yielding elite rice cultivar used for backcrossing and flowers in early August; Akitakomachi is commonly grown in Akita, Japan, and flowers in late July (1 week before Akita 63); and Nipponbare is the model japonica rice cultivar that flowers in late August in Akita, Japan (Table 1). Locations of SNPs found in *Hd1* sequences and the resulting amino acid substitutions are summarized in Figure 2a. The results showed that the *Hd1* sequence of Akita 63 was identical to that of Akitakomachi but different from the *Hd1* sequences of Nipponbare and Kinmaze (Figure 2b–d). The *Hd1* sequence of Kinmaze was also different from that of Nipponbare. In addition, the *Hd1* sequences of Akita 63 and Akitakomachi were 4807 bp in length, while that of Kinmaze was 4850 bp. The *Hd1* of Nipponbare was 4814 bp in length and contained two exons (1325–2152 bp and 2790–3149 bp) (Figure 2a) [4]. The *Hd1* of Akita 63, Akitakomachi, and Kinmaze carried a cytosine to thymine polymorphism at the 1640th nucleotide relative to the *Hd1* of Nipponbare, resulting in a histidine to tyrosine substitution (Figure 2a,b,d). In addition, the *Hd1* of Akita 63, Akitakomachi, and Kinmaze harbored 36 nucleotide insertions between the 1657th and 1658th nucleotides, resulting in 12 amino acid insertions between the 110th and 111th amino acid residues, compared with Nipponbare (Figure 2a,b,d). The remaining *Hd1* sequence in Kinmaze was the same as that in Nipponbare. Therefore, Kinmaze was predicted to produce a functional Hd1 protein (Figure 2b–d). On the contrary, the *Hd1* of Akita 63 and Akitakomachi contained 43 additional nucleotide deletions between the 2032nd and 2074th nucleotides compared with the *Hd1* of Nipponbare, resulting in a frame shift at the 236th amino acid and a premature stop codon at the end of exon 1 (Figure 2a–d). Akita 63 and Akitakomachi theoretically produced only 259 of the 407 amino acids of the Hd1 protein (Figure 2d), although the truncated protein could be degraded. Therefore, Akita 63 and Akitakomachi were speculated to produce a nonfunctional hd1 protein.

### 2.2. Genotyping and Western Blotting of Rice Accessions with Different Hd1 and SSIIa Alleles

**Figure 3:**
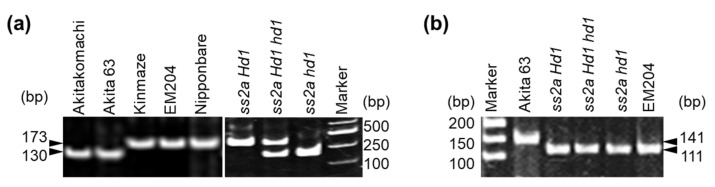
PCR-based screening of rice accessions with variable *Hd1* and *SSIIa* genotypes. (**a**) Screening of *Hd1* alleles using #5 and #12 PCR primers listed in Table S1. Lines with the *hd1* allele generated 130 bp PCR products, while those with the *Hd1* allele generated 173 bp PCR products; the heterozygous (*Hd1 hd1*) line produced both PCR products. (**b**) Screening of the *ss2a* allele. Lines carrying the *ss2a* allele showed 111 bp fragment after digesting with *Bgl*II, whereas Akita 63 (harboring the *ss2a^L^* allele) showed a 141 bp band (undigested by *Bgl*II).

PCR markers for *Hd1* have been generated for the selection of the early-flowering trait in rice cultivars such as KantoHD1 [45] and Milky Summer [46], which were generated via the introduction of the nonfunctional *hd1^Kas^* allele from Kasalath. It is important to note that although both Kasalath and Akita 63 flower at the same time (early August in Akita, Japan), the *hd1^Kas^* allele of Kasalath is different from that of Akita 63 (Table 1, Figure 2d). Therefore, such selection markers would not be applicable to Akita 63 (Figure 2d). To distinguish the *hd1*, *Hd1 hd1*, and *Hd1* seedlings from the NILs generated by crossing EM204 and Akita 63, a new molecular marker was generated (Figure 3a, Table S1 and Figure S1). Early-flowering lines with the *hd1* allele (such as Akita 63, Akitakomachi, and *ss2a hd1*) generated 130 bp PCR products, whereas late-flowering lines with the *Hd1* allele (such as Nipponbare, Kinmaze, EM204, and *ss2a Hd1*) generated 173 bp PCR products (Figure 3a). Both 173 and 130 bp PCR products were detected in the heterozygous (*Hd1 hd1*) line (Figure 3a). The PCR products exhibited clear differences in migration patterns, thus enabling the distinction among the *Hd1*, *Hd1 hd1*, and *hd1* lines (Figure 3a). Presence of the *ss2a* allele was confirmed via the derived cleaved amplified polymorphic sequence (dCAPS) marker (Figure 3b, [43]); the 141 bp PCR product amplified from Akita 63 was not digested by *Bgl*II, while that amplified from *ss2a Hd1*, *ss2a Hd1 hd1*, *ss2a hd1*, and EM204 was digested into 111- and 30-bp products by *Bgl*II.

**Figure 4:**
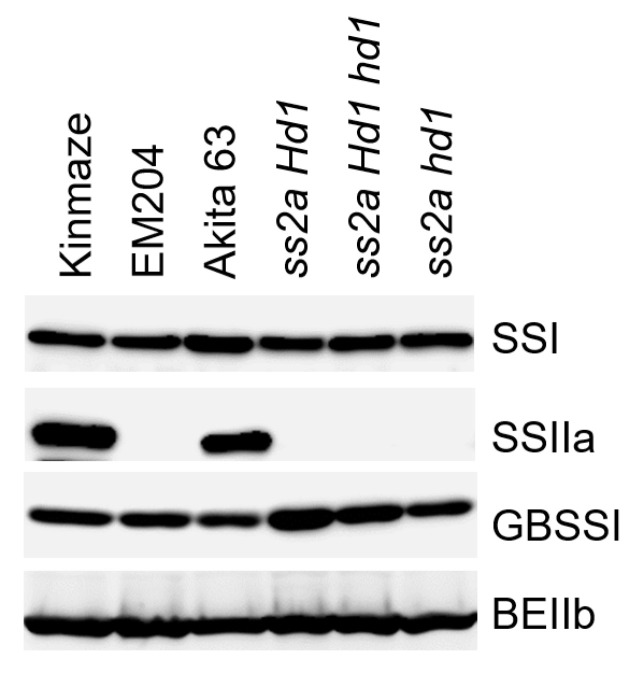
Western blotting analysis of total protein extract prepared from the mature seeds of different rice accessions. Starch biosynthetic enzymes were detected using the corresponding antibodies.

Western blotting of the total protein extracted from mature seeds using anti-SSIIa antibody confirmed the absence of SSIIa in *ss2a Hd1*, *ss2a Hd1 hd1*, *ss2a hd1*, and EM204 and the presence of SSIIa in Kinmaze and Akita 63 (Figure 4). The *Hd1* and *SSIIa* genotypes of rice accessions used in this study are summarized in Table 1. Differences in protein levels of SSI, GBSSI, and BEIIb are explained below (Section 2.4 and Section 2.5).

### 2.3. Effect of Hd1 Alleles on the Agricultural Traits of NILs

**Figure 5:**
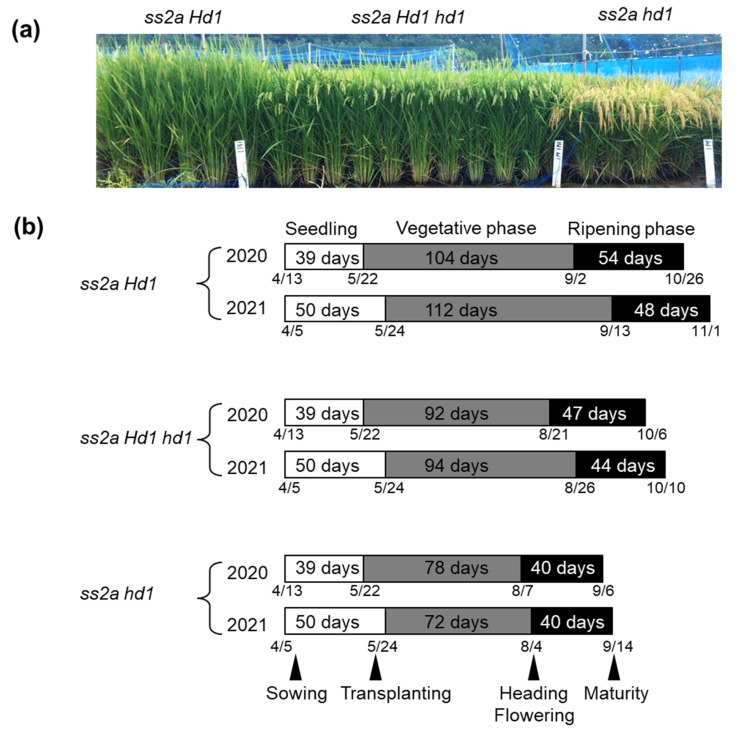

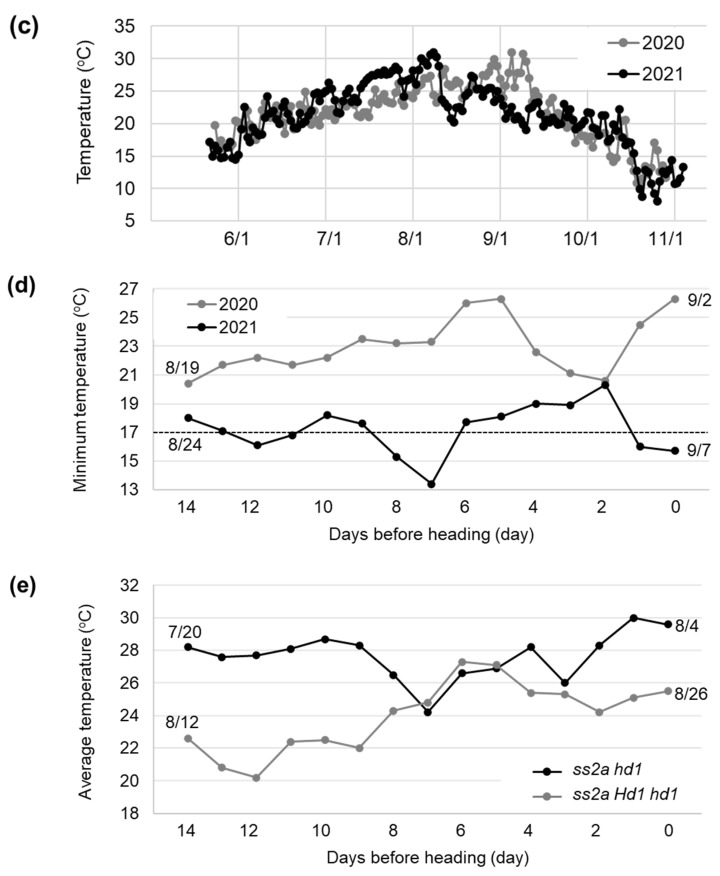
Comparison of the heading dates of *ss2a Hd1*, *ss2a Hd1 hd1*, and *ss2a hd1*. (**a**) Photo of NILs showing the differences in their heading dates. Note that *ss2a hd1* is mature, *ss2a Hd1 hd1* is at the mid-developmental stage, and *ss2a Hd1* is still flowering. (**b**) Differences among rice NILs in the number of days to heading and to maturity. Numbers (month/day) below the ribbon represent the actual dates of sowing, transplanting, heading, flowering, and maturity. (**c**) Average day temperature during the period from the end of May (transplanting) to the beginning of November (harvesting) in 2020 (gray) and 2021 (black). (**d**) Minimum temperature for 2 weeks before the heading date of *ss2a Hd1* in 2020 (gray) and 2021 (black). Dashed line indicates the threshold temperature (17 °C) that reduces the fertility rate. (**e**) Average temperature for 2 weeks before the heading date of *ss2a hd1* (black) and *ss2a Hd1 hd1* (gray) in 2021.

**Figure 6:**
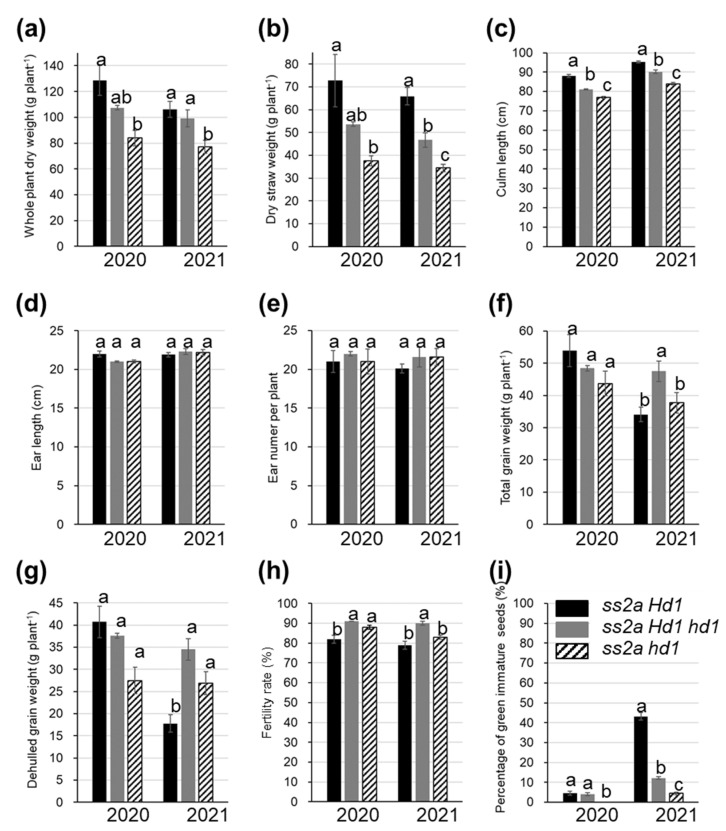
Agricultural traits of *ss2a Hd1* (black), *ss2a Hd1 hd1* (gray), and *ss2a hd1* (stripe) NILs. (**a**) Whole-plant dry weight, (**b**) dry straw weight per plant, (**c**) culm length, (**d**) ear length, (**e**) ear number per plant, (**f**) total grain weight per plant, (**g**) dehulled grain weight per plant, (**h**) fertility rate, (**i**) percentage of green immature seeds. Data represent mean ± standard error (SE). The three bars on the left represent data from 2020, and those on the right represent data from 2021. Data collected during the same harvest year were statistically analyzed via the Tukey-Kramer method (*p* < 0.05). The number of *ss2a Hd1*, *ss2a Hd1 hd1*, and *ss2a hd1* plants was 9, 20, and 8, respectively, in 2020, and 20 plants of each line were analyzed in 2021. Different lowercase letters above bars indicate significant differences.

The agricultural traits of *ss2a Hd1*, *ss2a Hd1 hd1*, and *ss2a hd1* NILs were examined over 2 years (Figure 5 and Figure 6, Tables S2 and S3). The three NILs were germinated or transplanted on the same respective dates and grown in the same paddy field under the same growth conditions. The heading dates of *ss2a Hd1*, *ss2a Hd1 hd1*, and *ss2a hd1* lines were remarkably different (Figure 5a,b). Although the actual heading dates of NILs slightly differed between the two years, they showed the same trend (Figure 5b, Table 1). The *ss2a hd1* line showed the earliest heading date (early August; August 4 or 7), followed by *ss2a Hd1 hd1* (late August; August 21 or 26) and *ss2a Hd1* (early September; September 2 or 13). The flowering period of individual plants of the same genotype was well synchronized; plants of the same genotype flowered within 2–3 days. In addition, the seed development and maturation period showed the order *ss2a hd1* < *ss2a Hd1 hd1* < *ss2a Hd1*, and the *ss2a hd1*, *ss2a Hd1 hd1*, and *ss2a Hd1* lines took 40, 44–47, and 48–54 days, respectively, to reach maturity after heading (Figure 5b). Only the *ss2a Hd1* line was prematurely harvested on 1 November 2021, since no further seed development was expected because of the arrival of winter (Figure 5c). The vegetative phase of lines *ss2a Hd1*, *ss2a Hd1 hd1*, and *ss2a hd1* was 104–112, 92–94, and 72–78 days, respectively (Figure 5b), and correlated well with the whole-plant dry weight, dry straw weight, plant height, and culm length (Figure 6a–d, Tables S2 and S3). The longer the vegetative period, the longer the culm and the heavier the straw weights. However, the duration of the vegetative phase did not influence the length and number of ears (Figure 6e,f, Tables S2 and S3).

The total grain weight and dehulled grain weight of *ss2a Hd1 hd1* were greater than those of *ss2a hd1* (Figure 6f,g). While those of *ss2a Hd1* differed between the 2 years, those values of *ss2a Hd1* were greater than those of *ss2a Hd1 hd1* in 2020 but lower than those of *ss2a hd1* in 2021. Some correlation was detected between plant biomass and grain yield; the higher the biomass, the better the yield, as long as the temperature during seed development remained optimal (Figure 5c and Figure 6a,b,f,g). Reduction in the grain yield of *ss2a Hd1* in 2021 was likely caused by low temperature from mid-August to early September (Figure 5c,d). This delayed the heading date, which prolonged seed development and reduced starch synthesis, thus increasing the number of premature grains (Figure 6i). Low average day temperature is also known to prolong the seed maturation period [47]. In fact, the *ss2a Hd1* plants did not fully reach maturity in 2021 (Figure 6g, Tables S2 and S3). Although the effect of this phenomenon was minor, low temperature also led to reduced fertility rate (Figure 5d and Figure 6h). Studies show that fertility rate declines when the minimum temperature remains under 17 °C for 2 weeks before the heading date [48] and when the temperature is too high [49]. These findings are consistent with the lower fertility rates of *ss2a Hd1* and *ss2a hd1* than that of *ss2a Hd1 hd1* (Figure 5e and Figure 6h). Therefore, the functional *Hd1* allele is unsuitable for rice cultivars grown in high-latitude areas for the maintenance of stable grain quality and yield. However, if an increase in plant biomass is desired, especially for rice cultivars utilized as feed (straw) or for ethanol production, the functional *Hd1* allele is necessary for prolonging the vegetative phase.

The average weight of one dehulled seed of EM204 was only 16.5 mg [43], which is approximately 55% of that of *ss2a Hd1*. Therefore, the yield of *ss2a Hd1*, *ss2a Hd1 hd1*, and *ss2a hd*1 lines generated in the present study was greatly improved, owing to backcrossing with the high-yielding parental line Akita 63.

### 2.4. Effect of Hd1 Alleles on Apparent Amylose Content and GBSSI Expression Level

Apparent amylose content affects texture of cooked rice and rice products [50,51], and the abundance of GBSSI, which is responsible for amylose synthesis, is affected by the temperature during seed development [30]. Since the temperature during seed development varied considerably among NILs, depending on their heading dates (Figure 5c, Table S4), the apparent amylose content was measured via gel filtration chromatography using a series of single HW-55S and triple HW-50S Toyopearl columns (Table 2, Figure S2). Amylose was eluted in fraction I, and the long and short chains of amylopectin were eluted in fractions II and III, respectively (Figure S2).

Apparent amylose content of Akita 63 (17–18%) was relatively low (Table 2) compared with that of Kinmaze (22%) [43,52]. This is partly because Akita 63 flowered in early August when the temperature was high (average day temperature = 25–30 °C) during seed development, while Kinmaze flowered in early September under lower temperature (average day temperature = ~20 °C). Although the apparent amylose content of EM204 (24%) was higher than that of Kinmaze, both rice accessions flowered at a similar time (early September; average day temperature = ~20 °C). Therefore, the absence of SSIIa resulted in an increase of apparent amylose content in the Kinmaze background. Similarly, the amylose contents of all three NILs were significantly higher than that of Akita 63, as determined using a pairwise *t*-test (Table 2).

To determine whether the different heading dates of NILs affect apparent amylose content in the absence of SSIIa in the Akita 63 background, the apparent amylose contents of *ss2a Hd1*, *ss2a Hd1 hd1*, and *ss2a hd1* were compared (Table 2, Figure S2). The results showed that the apparent amylose contents of *ss2a Hd1*, *ss2a Hd1 hd1*, and *ss2a hd1* were 27.0%, 25.1%, and 22.1%, respectively, in 2020, and 28.0%, 26.6%, and 24.7%, respectively, in 2021 (Table 2, Figure S2). Thus, the amylose content of *ss2a Hd1* was the highest among the three lines and was significantly higher than that of *ss2a hd1*. We found that the earlier the heading date, the higher the seed development temperature and the lower the amylose content (Table 2, Table S2 and S3, Figure S2). The apparent amylose content of *ss2a hd1* was 3–5% lower than that of *ss2a Hd1* and 4–8% higher than that of Akita 63 (Table 2). This suggests that loss of SSIIa mitigates the reduction in amylose content, even if the temperature during seed development is high.

To determine whether apparent amylose content is correlated with the GBSSI protein level, we performed western blotting of NILs (Figure 4). The amount of GBSSI protein showed a strong correlation with the apparent amylose content (Figure 4). Additionally, the GBSSI protein was the least abundant in Akita 63, and the level of GBSSI in *ss2a Hd1* was greater than that in *ss2a hd1* (Figure 4).

### 2.5. Effect of Hd1 Alleles on Amylopectin Structure

**Figure 7:**
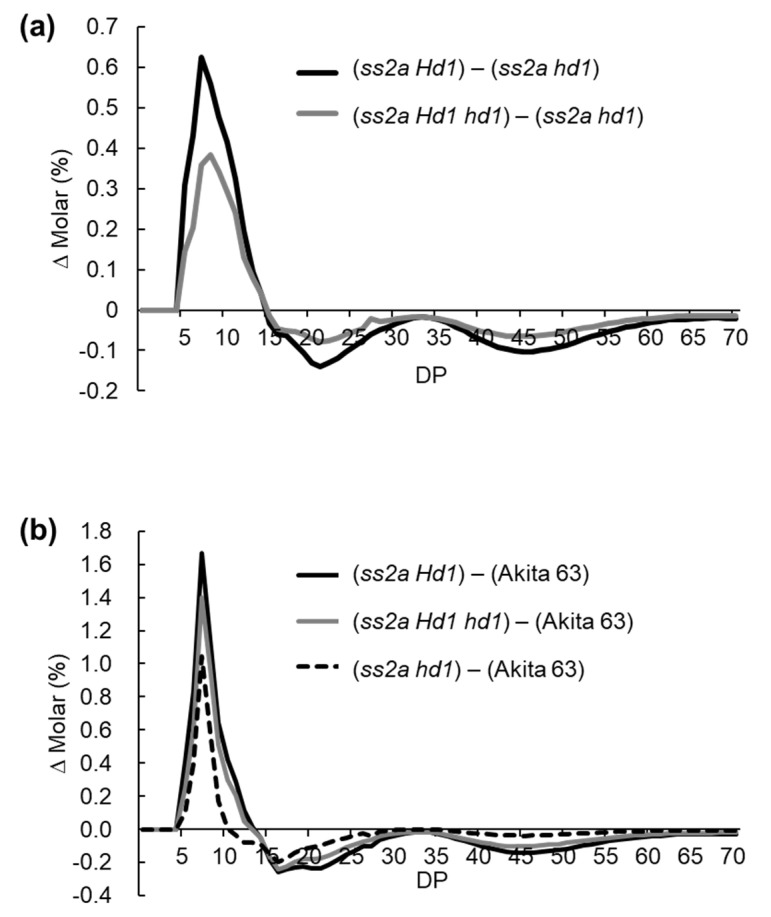
Differences in the amylopectin branch structure of NILs. (**a**,**b**) Subtraction curves showing the effects of *Hd1* alleles on amylopectin branch structure (**a**) and the effect of the loss of SSIIa on amylopectin structure (**b**). Each panel shows one typical representative data set of at least three replications. Data shown here were obtained from samples harvested in 2021, and the data of samples harvested in 2020 are shown in Figure S3.

The ratio of short amylopectin chains to long amylopectin chains (eluted in fraction III and fraction II, respectively, via gel filtration chromatography) was higher in *ss2a Hd1* than in *ss2a hd1* (Table 2). Therefore, the detailed amylopectin branch structure was analyzed via capillary electrophoresis using debranched starch purified from mature rice seeds (Figure 7 and Figure S3). The differences in amylopectin structure were shown as a differential curve.

To reveal the effect of heading date on amylopectin structure, values of chain length distribution of *ss2a hd1* were subtracted from those of *ss2a Hd1* or *ss2a Hd1 hd1* (Figure 7 and Figure S3). The results showed that the number of short amylopectin chains (DP < 14) was larger in *ss2a Hd1* and *ss2a Hd1 hd1* seeds than in *ss2a hd1* seeds harvested in both years. While the number of long amylopectin chains (DP ≥ 15) was larger in *ss2a hd1* than in *ss2a Hd1* and *ss2a Hd1 hd1* (Figure 7a and Figure S3a). The degree of difference was greater in *ss2a Hd1* than in *ss2a Hd1 hd1* (Figure 7a and Figure S3a). The reason why *ss2a hd1* seeds contained fewer short amylopectin chains and more long amylopectin chains was probably because of a slight decrease in BEIIb levels in *ss2a hd1*, as shown via western blotting (Figure 4).

To reveal the effect of the loss of SSIIa on amylopectin structure, values of chain length distribution from Akita 63 were subtracted from those of *ss2a Hd1*, *ss2a Hd1 hd1*, or *ss2a hd1*. All three NILs, which lacked SSIIa, showed similar trends, i.e., a considerable increase in short amylopectin chains with DP 5–10 and a decrease in intermediate amylopectin chains with DP 12–24 (Figure 7b and Figure S3b). These results are consistent with the role of SSIIa, which synthesizes intermediate chains [43].

### 2.6. Effect of Hd1 Alleles on the Thermal Properties of Starch

The gelatinization temperature of starch depends on the number of amylopectin branches with DP ≤ 24 [53,54]. An increase in short amylopectin branches lowers the gelatinization temperature [43], while an increase in long amylopectin branches raises the gelatinization temperature [52]. Therefore, we measured the gelatinization temperature of starch in *ss2a Hd1*, *ss2a Hd1 hd1*, or *ss2a hd1* using differential scanning calorimetry and compared the results with the gelatinization temperature of starch in Akita 63 (Table 3).

The gelatinization temperature of lines lacking SSIIa (*ss2a Hd1*, *ss2a Hd1 hd1*, and *ss2a hd1*) was lower than that of Akita 63. To precisely evaluate the effect of the absence of SSIIa on starch gelatinization temperature, the gelatinization temperatures of starch in *ss2a hd1* and Akita 63 were compared since both rice accessions flowered in early August. The peak gelatinization temperature of *ss2a hd1* was 1.3–4.8 °C lower than that of Akita 63, although the heading dates of both these accessions were essentially the same. This is because *ss2a hd1* (owing to the loss of SSIIa) contained a higher number of short amylopectin chains with DP < 10 and lower number of chains with DP ≥ 10 than Akita 63 (Figure 7b). This suggests that the loss of SSIIa lowers the gelatinization temperature of starch, even under high temperature during seed development.

In addition, the peak gelatinization temperature of NILs followed the order *ss2a hd1* > *ss2a Hd1 hd1* > *ss2a Hd1*, although exact values of each line differed between the years (Table 3). This trend of the peak gelatinization temperature of NILs may be explained by differences in the chain length distribution of amylopectin among the NILs: the number of amylopectin chains with DP < 15 showed the order *ss2a Hd1* > *ss2a Hd1 hd1* > *ss2a hd1* and that of amylopectin chains DP > 15 followed the order *ss2a Hd1* < *ss2a Hd1 hd1* < *ss2a hd1* (Figure 7a). This suggests that gelatinization temperature is affected by the heading date: the higher the temperature during seed development, the higher the gelatinization temperature of starch, even in the absence of SSIIa (Figure 5, Table 3 and Table S4).

## 3. Discussion

### 3.1. SNPs in Hd1

In this study, SNPs responsible for the differences in the heading dates of Kinmaze (the parental line of the *ss2a* null mutant EM204), Akita 63, and Akitakomachi were identified. Furthermore, the precise effects of different heading dates, determined by *Hd1*, *Hd1 hd1*, and *hd1*, on the agricultural traits and starch properties of rice were evaluated in NILs (lacking SSIIa) generated using Akita 63, an elite rice cultivar, as the recurrent parent. Sequencing analyses revealed that Akita 63 carries a loss-of-function *hd1* allele, while Kinmaze harbors a functional *Hd1* allele. The heading date of *ss2a hd1* was the earliest and 72–78 days after transplanting. Heading dates of *ss2a Hd1 hd1* and *ss2a Hd1* were 14–19 and 26–40 days later than those of *ss2a hd1*, respectively. These differences in the heading dates of *ss2a Hd1*, *ss2a Hd1 hd1*, and *ss2a hd1* were likely caused by the different *Hd1* alleles, although several other genes are also involved in determination of the heading date (Figure 1).

In addition, analyses of *Hd1* gene sequences using the basic local alignment search tool (BLAST; https://blast.ncbi.nlm.nih.gov/Blast.cgi, accessed on 20 June 2019) and the alignment of Hd1 amino acid sequences revealed that the *hd1* allele of Akita 63 and Akitakomachi is identical to that of the HS66 mutant (AB041841; [4]) and Sasanishiki (AB433218) (Figure S4a) but different from that of Kasalath (AB041839; [4], Figure 2d), Ginbouzu (AB041840; [4]), and Koshihikari (AB375859; [6]) (Figure S4b). The *Hd1* allele of Koshihikari is identical to that of Nipponbare, while Ginbouzu shares the same *Hd1* sequence as Kinmaze (MK449352; this study), Hoshinoyume (AB353276; [7]), and Hayamasari (AB353275; [7]) (Figure S4c). The PCR marker generated in this study (Table S1, Figure S1) as well as other PCR markers generated by Mo et al. [18] will serve as useful tools for determining the different types of *Hd1* alleles, which will accelerate the breeding of new rice cultivars with different heading dates. Different *Hd1* alleles have already been utilized to distribute the workload of the peak harvesting hours. For example, low-amylose rice lines harboring the *Wx^mq^* gene, such as Milky Summer, Milky Queen, and Milky Autumn, are grown in the central to southern parts of Japan (https://www.naro.go.jp/publicity_report/press/laboratory/nics/079175.html, accessed on 28 June 2022). The choice of different *Hd1* alleles should be carefully considered, depending on the application (yield increase, starch property, or workload distribution).

### 3.2. Effect of Hd1 Alleles on Grain Yield

Differences in the heading date impacted the agricultural traits of NILs (Figure 6, Tables S2 and S3). The total grain yield of *ss2a Hd1 hd1* tended to be higher than that of *ss2a hd1*, although it was statistically insignificant due to a statistics outlier, while the total grain yield of *ss2a Hd1* varied depending on the year (Figure 6, Tables S2 and S3). The percentages of green immature grains were lowest in *ss2a hd1* and highest in *ss2a Hd1* (Figure 6, Tables S2 and S3). Presence of the *hd1* allele enabled efficient grain filling by promoting flowering at the appropriate temperature for starch biosynthesis during seed development, thus minimizing the time required for seed maturation and desiccation. However, because of the short vegetative period, the amount of stored photosynthetic products to be translocated from the culm might be decreased, which may lead to reduced yield. Thus, the heading date of *ss2a Hd hd1* seemed the most suitable for cultivation in Akita (Japan) as it showed stable high-level production of grains, judging from the limited data obtained under the extreme temperature conditions in 2020 and 2021, although the heterozygous allele (*Hd1 hd1*) would not be appropriate for commercial rice production as it would segregate in subsequent generations. The *ss2a hd1* NIL is also suitable for cultivation in northern Japan because the percentage of green immature grains of this genotype was the lowest, although its yield could be improved further. Increase in grain yield while maintaining seed quality should be possible by finetuning the combinations of other genes involved in the determination of the heading date, to ensure that the rice flowers in mid-August.

The *ss2a Hd1* NIL was not suitable for grain production because the heading date was too late and risked the early arrival of winter during seed development, which could lead to large yield differences between years. Moreover, if the heading date is delayed because of low temperature in August, there is a high chance that seed development may not be completed in time, resulting in drastic yield losses. However, *ss2a Hd1* showed the highest culm length and straw dry weight. Therefore, use of the *Hd1* allele would be beneficial for increasing the plant biomass, which could be used as feed for livestock or as a raw material for bioethanol production. Farmers generally prefer to grow rice varieties with relatively shorter culm length to avoid lodging. Short culm produces less waste, requires less fertilizer, and improves work efficiency. Therefore, cultivars with suitable *Hd1* alleles should be carefully considered, depending on whether the ultimate goal is to harvest grains or whole plants. The latitude and altitude of the planting area should also be taken into account when selecting rice cultivars with different *Hd1* alleles. Since *Hd1* functions by repressing heading under long days and promoting heading under short days [5,14,16], the effects of *Hd1* at different latitudes are expected to differ. The presence of the *hd1* allele likely prevents premature heading of rice plants grown near the equator and helps increase the tiller and ear numbers before transitioning to the reproductive phase. Growing *ss2a Hd1*, *ss2a Hd1 hd1*, and *ss2a hd1* genotypes at different latitudes and temperatures will provide additional useful information for achieving high yields in the respective regions.

### 3.3. Effect of Hd1 Alleles on Starch Structure

High temperature during seed development reduces apparent amylose content by reducing the abundance of GBSSI and increases the gelatinization temperature of starch by decreasing the abundance of BEIIb, thus affecting the quality of rice [28,30,40,42]. Compared with the effects of high temperature, the loss of SSIIa activity has opposite effects; the *ss2a* mutant shows higher apparent amylose content and lower gelatinization temperature compared with its parental line [43]. To reveal whether the loss of SSIIa can mitigate the above-described effect on starch under high temperature during seed development, the starch properties of *ss2a Hd1*, *ss2a Hd1 hd1*, and *ss2a hd1* NILs were evaluated since temperatures during the seed development of these lines were low, medium, and high, respectively, because of differences in their heading dates (Figure 5c and Table S4). The apparent amylose content of *ss2a hd1* was 22.1–24.7%, which was lower than that of *ss2a Hd1 hd1* (23.2–26.6%) and *ss2a Hd1* (27.0–28.0%) but higher than that of Akita 63 (17.1–18.1%) (Table 2). Nonetheless, both *ss2a hd1* and Akita 63 flowered at almost the same time and possessed an identical genetic background, except SSIIa. Therefore, the loss of SSIIa increased the apparent amylose content even if seed development occurred under high temperature. Increasing the apparent amylose content of rice grains can be used as one of the breeding strategies for increasing the health benefit of rice, since high apparent amylose content elevates the resistant starch content [52,55]. The *Hd1 hd1* and *Hd1* alleles are beneficial for increasing the apparent amylose content because these alleles delay flowering and facilitate seed development under cooler temperatures. However, to achieve high yield and avoid the risk of the early arrival of winter, heading dates should be no later than late August, especially if the rice is grown in the northern area of Japan.

### 3.4. Effect of Hd1 Alleles on Starch Gelatinization Temperature

The *ss2a hd1* NIL possessed a higher number of short amylopectin chains (DP < 15) than Akita 63, and its gelatinization temperature (57 °C) was lower than that of Akita 63 (62.0 °C) but higher than that of *ss2a Hd1 hd1* (55.5 °C) and *ss2a Hd1* (52.3 °C) (Figure 7, Table 3). One of the reasons why the gelatinization temperature of *ss2a hd1* was higher than that of *ss2a Hd1 hd1* and *ss2a hd1* might be the relatively lower abundance of the BEIIb protein under high temperature, which increased the number of long amylopectin branches (Figure 4). The balance between amylopectin branch generation and removal is important for controlling amylopectin structure, and loss of BEIIb can be mitigated by the additional loss of isoamylase 1 [56]. Therefore, reduction in the isoamylase 1 level may be one way to counterbalance the reduction in BEIIb level under high temperature during seed development. Alternatively, delaying the heading date of *ss2a hd1* offers a more practical way. Possible target genes for delaying the flowering time of *ss2a hd1* are *Ghd7* and *OsPRR37*, since combinations of the presence or absence of these genes and that of *Hd1* alleles allow the heading date of rice to be further finetuned [57,58]. Rice with a low gelatinization temperature is expected to retrograde slowly and be tasty. Thus, introduction of the *ss2a* allele into rice lines cultivated near the equator (with high temperature during seed development) may improve the quality of rice and rice products produced in tropical regions. Analysis of the retrogradation properties of *ss2a Hd1*, *ss2a Hd1 hd1*, and *ss2a hd1* lines will provide additional information for the use of these NILs in the food industry.

## 4. Materials and Methods

### 4.1. Plant Materials

Rice (*Oryza sativa* L.) *ss2a* mutant, EM204, was previously isolated from the NMU-mutagenized populations of the wild-type japonica cultivar, Kinmaze, which flowers late (early September) at high latitude [43]. EM204 harbors a mutation at the last nucleotide of intron 5, which inhibits splicing and results in the deficiency of 15 amino acids [43]. EM204 was backcrossed twice with the early-flowering, high-yielding elite japonica rice cultivar, Akita 63 [44]. The resulting F_1_ seedlings were grown and self-pollinated to obtain the F_2_ progeny. DNA was isolated from F_2_ seedlings, and genotyping was performed as described previously [43]. The *ss2a Hd1 hd1* line was self-pollinated to obtain *ss2a Hd1*, *ss2a Hd1 hd1*, and *ss2a hd1* NILs. Theoretically, 87.5% of the genome in these three NILs was derived from Akita 63. Akitakomachi was obtained from Akita Prefectural Agricultural Experiment Station, Akita, Japan, and Kasalath and Nipponbare were obtained from the Genebank, National Agricultural and Food Research Organization, Tsukuba, Japan. All rice lines were grown in an experimental paddy field of Akita Prefectural University during the summer under natural light conditions.

### 4.2. Sequencing of the Hd1 Gene

Genomic DNA was isolated from leaves of Akita 63, Akitakomachi, and Kinmaze. Approximately 3 cm of young leaf was powdered with liquid nitrogen using a Multi-beads Shocker (Yasui Kikai, Osaka, Japan). The powder was extracted with 400 μL of 200 mM Tris-HCl, pH 7.5, 250 mM NaCl, 25 mM EDTA, 0.5% SDS. After centrifugation, 300 μL of the supernatant was mixed with an equal volume of isopropanol, let stand for 20 min or longer, and centrifuged. The DNA pellet was rinsed with 70% ethanol, dried, and resuspended in 25 μL of TE buffer containing 25 μL of 10 mM Tris-HCl, 1 mM EDTA. 1 μL of DNA was used for 10 μL PCR reaction. PCR amplification was carried out using the Quick Taq HS dye mix (TOYOBO, Osaka, Japan), dimethyl sulfoxide (DMSO; 5% final concentration), and sequence-specific primers (Table S1) under the following conditions: 94 °C for 2 min, and 38 cycles of 94 °C for 20 s, 50 °C for 20 s, and 68 °C for 20 s. The PCR products were sequenced at the Biotechnology Center in Akita Prefectural University, and the obtained sequences were aligned with that the *Hd1* gene of Nipponbare using Clustal Omega (https://www.ebi.ac.uk/Tools/msa/clustalo/, accessed on 20 June 2019) and analyzed using BLAST (https://blast.ncbi.nlm.nih.gov/Blast.cgi, accessed on 20 June 2019). The identified *Hd1* sequences were deposited in the NCBI GenBank database (https://www.ncbi.nlm.nih.gov/, accessed on 25 January 2019) under the following accession numbers: MK449350 (Akitakomachi), MK449351 (Akita 63), and MK449352 (Kinmaze).

### 4.3. Genotyping of Hd1 and SS2a Alleles

The *SSIIa* gene was genotyped as described [43]. To genotype the *Hd1* gene, PCR was performed using the Quick Taq HS dye mix (TOYOBO, Osaka, Japan), 5% DMSO, and sequence-specific primers (5′-GGCATGTATTTTGGTGAAGTCG-3′ and 5′-GTTGTCGTAGTACGAATTGTACCCGAC-3′) under the following conditions: 94 °C for 2 min, and 30 cycles of 94 °C for 20 s, 60 °C for 20 s, and 68 °C for 20 s. This enabled successful amplification, since the region was enriched in guanine and cytosine. PCR products were separated via electrophoresis on 15% acrylamide gel in 1× TBE buffer. The expected sizes of the PCR products were 170 bp for *Hd1* and 130 bp for *hd1*.

### 4.4. Field Experiments and Agricultural Traits

All rice lines were sown and transplanted on the same day, with a spacing of 20 cm between plants and 25 cm between rows. A total of 35 plants each of *ss2a Hd1* and *ss2a Hd1* genotypes, 100 plants of the *ss2a Hd1 hd1* genotype, and 20 plants each of the parental lines were grown according to the local agricultural practices. Heading date was recorded when 50% of plants of a given genotype initiated heading. Maturation date was recorded when 90% of the panicles turned yellow. Plant height and ear length were measured prior to harvesting. After 2 weeks of desiccation, whole-plant dry weight and total grain weight were measured, and dry straw weight was calculated by subtracting the total grain weight from the whole-plant dry weight. Total grain weight was measured including empty seeds. Grains were dehulled and sieved through a mesh with 1.9 mm pore size using a sieving machine, TEST Grain Selector (TWSB, Satake, Tokyo, Japan), and the weight of grains above 1.9 mm thickness and width was measured as total dehulled grain weight. Fertility rate was calculated by counting and subtracting the number of empty seeds from the total number of seeds. Quality of brown rice was analyzed using the VIRGO Rice Grain Selector (ES-V; Shizuoka Seiki, Shizuoka, Japan) by detecting the green premature seeds. Data were obtained in 2020 and 2021.

### 4.5. Meteorological Data

Meteorological data for 2021 were obtained from the Japan Meteorological Agency. Daily temperature data were extracted, and average temperature during seed development was calculated.

### 4.6. Western Blot Analysis

Three mature seeds of each rice genotype were ground to a fine powder, and total protein was extracted using 20 volumes (*w*/*v*) of buffer containing 125 mM Tris-HCl (pH 6.8), 8 M urea, 4% (*w*/*v*) SDS, 5% (*v*/*v*) β-mercaptoethanol, and 0.05% (*w*/*v*) bromophenol blue. After centrifugation, proteins in the supernatants were subjected to sodium dodecyl sulfate-polyacrylamide gel electrophoresis (SDS-PAGE) on 7.5% acrylamide gel and blotted onto a membrane. Membranes were incubated with the following primary antibodies: anti-SSI (1:3000 dilution [59]), anti-SSIIa (1:1000 dilution [60], anti-GBSSI (1:5000 [59]), and anti-BEIIb (1:5000 [35]). Subsequently, secondary antibody incubation and protein detection were performed as described previously [60].

### 4.7. Measurement of Apparent Amylose Content and Short to Long Chain Amylopectin Ratio

Starch was purified using the cold-alkaline method as described previously [61,62]. Purified starch was debranched using *Pseudomonas* isoamylase (Hayashibara, Okayama, Japan) and analyzed via gel filtration chromatography (Toyopearl HW-55S and HW-50S×3; Tosoh, Tokyo, Japan) [63,64,65]. Amylose (fraction I), long amylopectin chains (fraction II), short amylopectin chains (fraction III), and apparent amylose content were quantified as described previously [63,64,65].

### 4.8. Analysis of Amylopectin Structure

Debranched purified starch was fluorescently labeled and analyzed via capillary electrophoresis (P/ACE MDQ Plus Carbohydrate System; AB Sciex, Framingham, MA, USA), as described [66].

### 4.9. Measurement of Gelatinization Temperature

The thermal properties of purified starch were analyzed via differential scanning calorimetry (Seiko Instrument 6100; Seiko, Chiba, Japan) as described previously [59,67].

## 5. Conclusions

This study precisely evaluated the agricultural traits and starch properties of rice NILs (*ss2a hd1*, *ss2a Hd1 hd1*, and *ss2a Hd1*) lacking SSIIa and showing different heading dates, although the data were limited to two harvest years. These NILs were generated by crossing the elite rice cultivar Akita 63 (as the recurrent parent) with the *ss2a* null mutant EM204. Sequencing analyses revealed that Akita 63 carries a loss-of-function *hd1* allele, while Kinmaze (the parental line of EM204) possesses a functional *Hdl* allele. The *ss2a hd1* NIL was the first to initiate heading (early August), while the heading dates of *ss2a Hd1 hd1* and *ss2a Hd1* were approximately 2 and 4 weeks later, respectively, than that of *ss2a hd1*. The time required to reach maturity was the shortest in *ss2a hd1*, which reached maturation in mid-September, while the harvesting dates of *ss2a Hd1 hd1* and *ss2a Hd1* were approximately 4 and 6 weeks later, respectively, than that of *ss2a hd1*. Although *ss2a hd1* showed the lowest whole-plant dry weight, it also produced the lowest number of green immature seeds. Analyses of starch properties showed that the amylose content of *ss2a hd1* was lower than that of *ss2a Hd1*, but its gelatinization temperature was higher. Overall, this study provides useful information about the different heading dates, agricultural traits, and starch properties of rice accessions.

## Figures and Tables

**Table 1:** Summary of the *SSIIa* and *Hd1* genotypes and typical heading dates of rice accessions.

Rice Accession	*SSIIa* Genotype ^1^	*Hd1* Genotype	Heading Date ^2^
Nipponbare	*ss2a^L^*	*Hd1*	Late August
Kasalath	*SS2a*	*hd1^Kas^*	Early August
Akitakomachi	*ss2a^L^*	*hd1*	Late July
Akita 63	*ss2a^L^*	*hd1*	Early August
Kinmaze	*ss2a^L^*	*Hd1*	Early September
EM204	*ss2a*	*Hd1*	Early September
*ss2a Hd1*	*ss2a*	*Hd1*	Early September
*ss2a Hd1 hd1*	*ss2a*	*Hd1 hd1*	Late August
*ss2a hd1*	*ss2a*	*hd1*	Early August

^1^ Superscript L denotes leaky mutation present in the *SS2a* allele of wild-type japonica rice. ^2^ Typical heading dates from 2017 to 2021 in Akita, Japan. *hd1* allele of Kasalath is shown as *hd1^Kas^* to distinguish from that of Akitakomachi, Akita 63, and *ss2a hd1*.

**Table 2:** Apparent amylose content and ratio of short to long chain of amylopectin in different rice accessions.

Rice Accession	Apparent Amylose Content (%) ^1^		Ratio of Short to Long Chains of Amylopectin ^1^	
	2020	2021	2020	2021
Akita 63	18.1 ± 0.8	17.1 ± 0.4	2.4 ± 0.1	2.7 ± 0.1
*ss2a Hd1*	27.0 ± 0.6 a*	28.0 ± 0.1 a*	3.1 ± 0.0 a*	3.2 ± 0.0 a*
*ss2a Hd1 hd1*	25.1 ± 0.6 ab*	26.6 ± 0.6 ab*	2.6 ± 0.0 b	2.9 ± 0.0 b
*ss2a hd1*	22.1 ± 0.4 b*	24.7 ± 0.6 b*	2.4 ± 0.0 b	3.0 ± 0.0 ab

^1^ Apparent amylose content and the ratio of short to long chains of amylopectin were calculated from fraction I and fraction III/fraction II in Figure S2. Data represent the mean ± SE of three replicates. Different lowercase letters indicate significant differences among *ss2a Hd1*, *ss2a Hd1 hd1*, and *ss2a hd1* (Tukey-Kramer method; *p* < 0.05). Asterisk indicates significant differences relative to Akita 63 (*t*-test; *p* < 0.05).

**Table 3:** Peak gelatinization temperature (T_p_) of starch purified from rice grains harvested in 2020 and 2021, as analyzed via differential scanning calorimetry.

Rice Accession	T*p* (°C) ^1^	
	2020	2021
Akita 63	63.1 ± 0.0 a	62.0 ± 0.1 a
*ss2a Hd1*	50.3 ± 0.1 d	52.3 ± 0.2 d
*ss2a Hd1 hd1*	58.8 ± 0.1 c	55.5 ± 0.1 c
*ss2a hd1*	61.8 ± 0.1 b	57.2 ± 0.1 b

^1^ Data represent the mean ± SE of three replicates. Different lowercase letters indicate significant differences (Tukey-Kramer method; *p* < 0.05).

## Data Availability

The sequence data generated in this study are freely available from the NCBI GenBank database (accession numbers: MK449352.1, MK449351.1, MK449350.1).

## Supplementary Materials

The following supporting information can be downloaded at: https://www.mdpi.com/article/10.3390/ijms231810783/s1.
