# Supplementary material for: Effect of Heading Date on the Starch Structure and Grain Yield of Rice Lines with Low Gelatinization Temperature
Source: Int J Mol Sci. 2022 Sep 15;23(18):10783. doi: 10.3390/ijms231810783 (PMC9502985; doi:10.3390/ijms231810783)
Supplement: Supplementary file 1 [file ijms-23-10783-s001.zip › ijms-1907369-supplementary.pdf]

Supplementary Table S1: List of Primers Used for Sequencing.

#	Direction	Oligonucleotide sequence
1	Forward	5' GCAAAGATGAACAGAGGTGG 3'
2	Forward	5' GAGGCATATTTAACGTTATAGCAGGGC 3'
3	Forward	5' GGCTTAGTTCCATCTAAGAGATG 3'
4	Forward	5' CAAGAGCCATGCGAGGTAGAG 3'
*5	Forward	5' GGCATGTATTTTGGTGAAGTCG 3'
6	Forward	5' CAAGCTGCGATCTTTAGGCTG 3'
7	Forward	5' GCCAAGAGATCAGATGTGCAG 3'
8	Forward	5' GTGTCCACTGGCCAGATAGTATC 3'
9	Forward	5' GCACCAACCGCATATACAGG 3'
10	Reverse	5' GGATTCTGATGCTTGATTAATTCC 3'
11	Reverse	5' CTGGTCTGCCTAAAGATCAATTG 3'
*12	Reverse	5' GTTGTCGTAGTACGAATTGTACCCGAC 3'
13	Reverse	5' GCTTGCACCACATGGACAAGTCTATCG 3'

* Primers used for genotype screening of *hd1*.

Supplementary Table S2. Agricultural Traits of Rice NILs Grown in 2020.

Yield and yield components	*ss2a Hd1*	*ss2a Hd1 hd1*	*ss2a hd1*
Days to heading (days)	112	94	72
Days to maturation (days)	48	44	40
Plant height (cm)	110±0.4a	102±0.2b	98±0.6c
Culm length (cm)	88±0.7a	81±0.2b	77±0.5c
Ear length (cm)	22±0.4a	21±0.1a	21±0.2a
Ear number (No.)	21±1.4a	22±0.3a	21±1.6a
Whole plant dry weight (g)	128.5±11.5a	107.4±1.8ab	83.9±6.1b
Dry straw weight (g)	72.8±6.5a	53.6±1.0ab	37.6±2.2b
Total grain weight (g)	53.9±4.9a	48.5±0.8a	43.7±3.8a
Number of grains per 10 g (No.)	365.3±4.4b	354.7±0.6b	417.9±7.0a
Thousand grain weight (g)	27.4±0.3a	28.2±0.1a	24.0±0.4b
Percentages of fertile seeds (%)	82.2±1.6	91.0±0.2	87.7±2.1
Thousand dehulled-grain weight (g)	28.9±0.2a	27.0±0.0b	26.4±0.3b
Percentages of green immature grains (%)	4.6±1.0a	4.0±0.8a	0.1±0.0b

Average values are presented as mean ± standard error. n = 9, 20, and 8 for *ss2a Hd1*, *ss2a Hd1 hd1*, and *ss2a hd1*, respectively. Different letters (a-c) indicate significantly different from one another as determined by Tukey-Kramer method ($p < 0.05$).

Supplementary Table S3. Agricultural Traits of Rice NILs Grown in 2021.

**Yield and yield components**	***ss2a Hd1***	***ss2a Hd1 hd1***	***ss2a hd1***
Days to heading (days)	104	92	78
Days to maturation (days)	54	47	40
Plant height (cm)	117.2±0.5a	112.5±0.6b	106.2±0.6c
Culm length (cm)	95.3±0.5a	90.2±0.9b	84.0±0.7c
Ear length (cm)	21.9±0.3a	22.3±0.4a	22.2±0.4a
Ear number (No.)	20.1±0.6a	21.6±1.3a	21.6±1.1a
Whole plant dry weight (g)	106.0±6.2a	99.0±6.6a	77.1±4.9b
Dry straw weight (g)	65.9±3.9a	46.7±3.2b	34.4±1.7c
Total grain weight (g)	34.1±2.3b	47.5±3.2a	37.7±3.2b
Number of grains per 10 g (No.)	427.1±8.9a	347.6±2.4c	362.9±5.5b
Thousand grain weight (g)	23.6±0.5b	28.8±0.2a	27.7±0.4a
Percentages of fertile seeds (%)	78.7±2.0b	89.8±0.7a	82.7±1.2b
Thousand dehulled-grain weight (g)	29.8±0.2c	32.1±0.2b	33.4±0.3a
Percentages of green immature grains (%)	43.2±2.5a	12.2±1.1b	4.4±0.7c

Average values are presented as mean ± standard error (n=20). Different letters (a-c) indicate significantly different from one another as determined by Tukey-Kramer method ($p < 0.05$).

Supplementary Table S4. Average of daily temperature during the seed development.

Line	Maximum		Minimum		Day average	
	**2020**	**2021**	**2020**	**2021**	**2020**	**2021**
ss2a Hd1	21.4	21.7	13.4	13.1	17.0	17.0
ss2a Hd1 hd1	27.5	25.5	19.4	16.7	22.8	20.8
ss2a hd1	30.8	28.5	23.1	20.9	26.6	24.5

Meteoroidal dataset of the rice field was obtained from the Japan Meteorological Agency. Average temperature of daily high, low, and average were calculated for 2020 and 2021.

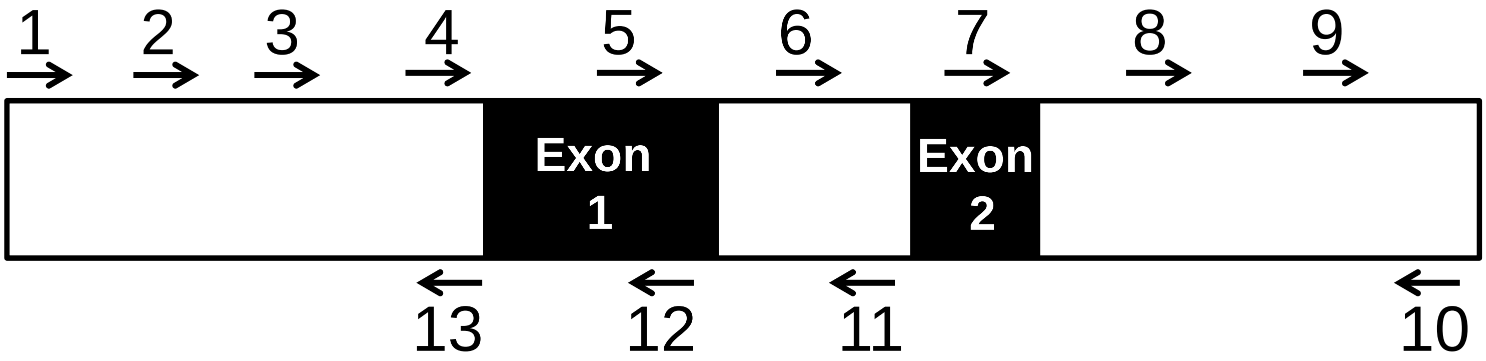

Supplementary Figure S1. Locations of primers used for sequencing the *Hd1* gene of rice accessions

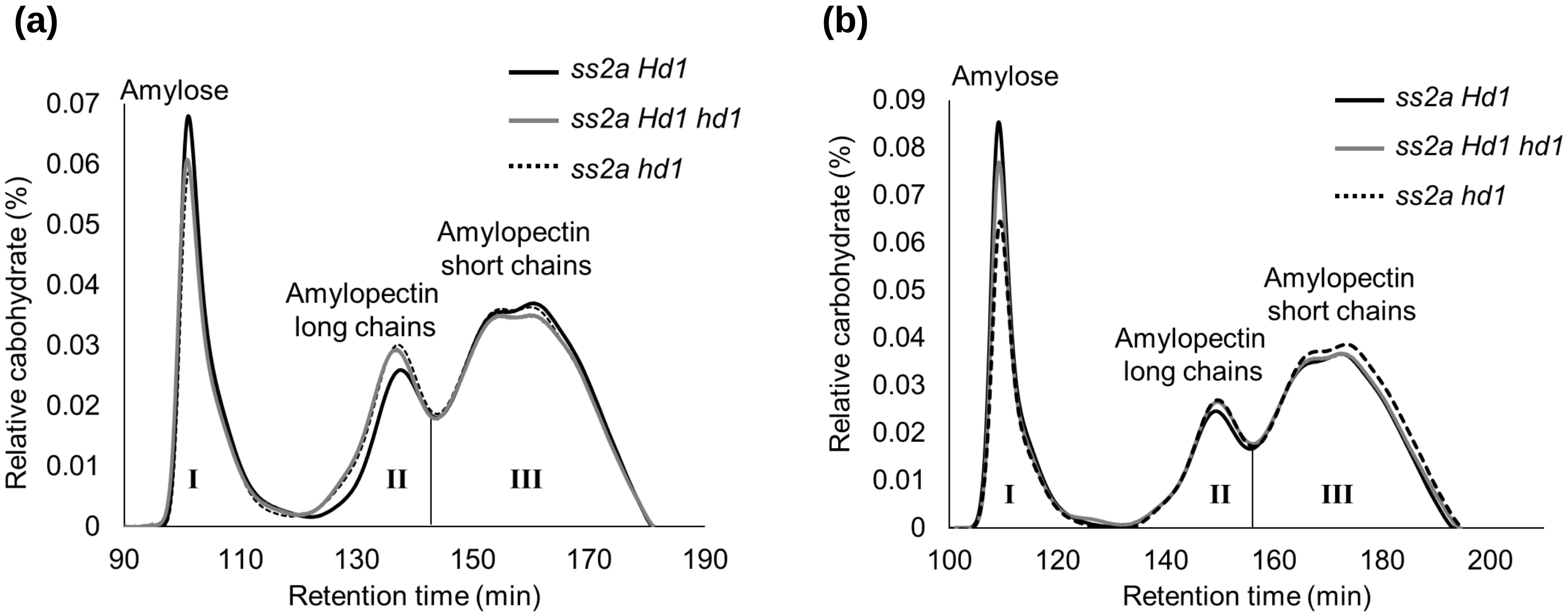

Supplementary Figure S2. Elution profiles rice accessions by gel filtration chromatography of debranched starch prepared from rice seeds harvested in (**a**) 2020 and (**b**) 2021. Three fractions (I, II, and III) were divided at the troughs of carbohydrate-content curve. Amylose elutes in fraction I, long chains of amylopectin branches elute in fraction II, and short chains of amylopectin branches elute in fraction III. Elution profiles are one of the representatives of at least three experiments. Exact values are shown in Table 4 and supplementary table 4.

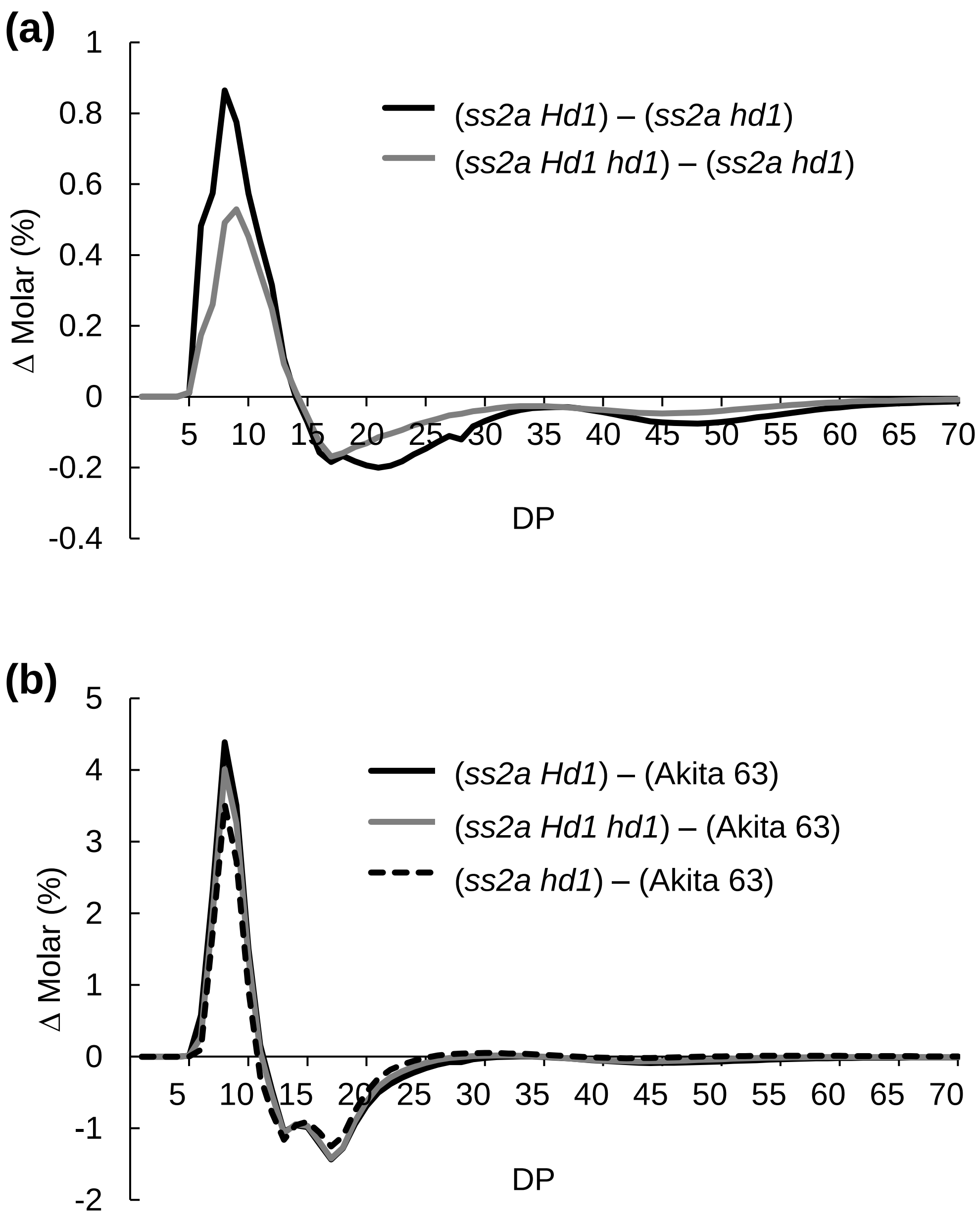

Supplementary Figure S3. Differences in amylopectin branch structure among rice NILs. Samples were prepared using rice seeds harvested in 2020. (**a**) The effects of the *Hd1* genotypes on amylopectin branch structure shown by subtraction curves as indicated. (**b**) The effect of loss of SSIIa on amylopectin structure shown by subtraction curves as indicated. Each panel shows one typical representative data set of at least three replications.

(a)

```
Akita63         MNYNFGGNVFDQEVGVGGEGGGGGEGSGCPWARPCDGCRAAPSVVYCRADAAYLCASCDA    60
HS66            MNYNFGGNVFDQEVGVGGEGGGGGEGSGCPWARPCDGCRAAPSVVYCRADAAYLCASCDA    60
Sasanishiki     MNYNFGGNVFDQEVGVGGEGGGGGEGSGCPWARPCDGCRAAPSVVYCRADAAYLCASCDA    60
                ************************************************************

Akita63         RVHAANRVASRHERVRVCEACERAPAALACRADAAALCVACDVQVYSANPLARRHQRVPV    120
HS66            RVHAANRVASRHERVRVCEACERAPAALACRADAAALCVACDVQVYSANPLARRHQRVPV    120
Sasanishiki     RVHAANRVASRHERVRVCEACERAPAALACRADAAALCVACDVQVYSANPLARRHQRVPV    120
                ************************************************************

Akita63         APLPAITIPATSVLAEAVVATATVLGDKDEEVDSWLLLSKDSDNNNNNNNNNDNDNNDNN    180
HS66            APLPAITIPATSVLAEAVVATATVLGDKDEEVDSWLLLSKDSDNNNNNNNNNDNDNNDNN    180
Sasanishiki     APLPAITIPATSVLAEAVVATATVLGDKDEEVDSWLLLSKDSDNNNNNNNNNDNDNNDNN    180
                ************************************************************

Akita63         NSNSSNNGMYFGEVDEYFDLVGYNSYYDNRIENNQDRQYGMHEQQEQQQQQQEMQKEFAE    240
HS66            NSNSSNNGMYFGEVDEYFDLVGYNSYYDNRIENNQDRQYGMHEQQEQQQQQQEMQKEFAE    240
Sasanishiki     NSNSSNNGMYFGEVDEYFDLVGYNSYYDNRIENNQDRQYGMHEQQEQQQQQQEMQKEFAE    240
                ************************************************************

Akita63         KEGSECVVMELWEQTRPPP          259
HS66            KEGSECVVMELWEQTRPPP          259
Sasanishiki     KEGSECVVMELWEQTRPPP          259
                *******************
```

Supplementary Figure S4. (**a**) Alignment of Hd1 in Akita 63, HS66, and Sasanishiki. Asterisks indicate identical amino acid sequences.

```
Akita63          MNYNFGGNVFDQEVGVGGEGGGGGEGSGCPWARPCDGCRAAPSVVYCRADAAYLCASCDA       60
Kasalath         MNYNFGGNVFDQEVGVGGEGGGGGEGSGCPWARPCDGCRAAPSVVYCRADAAYLCASCDA       60
Ginbouzu         MNYNFGGNVFDQEVGVGGEGGGGGEGSGCPWARPCDGCRAAPSVVYCRADAAYLCASCDA       60
Koshihikari      MNYNFGGNVFDQEVGVGGEGGGGGEGSGCPWARPCDGCRAAPSVVYCRADAAYLCASCDA       60
                 ************************************************************

Akita63          RVHAANRVASRHERVRVCEACERAPAALACRADAAALCVACDVQVYSANPLARRHQRVPV       120
Kasalath         RVHAANRVASRHERVRVCEACEQAPAALACRADAAALCVACDVQVHSANPLARRHQRVPV       120
Ginbouzu         RVHAANRVASRHERVRVCEACERAPAALACRADAAALCVACDVQVYSANPLARRHQRVPV       120
Koshihikari      RVHAANRVASRHERVRVCEACERAPAALACRADAAALCVACDVQVHSAN-----------       109
                 **********************:**********************:***

Akita63          APLPAITIPATSVLAEAVVATATVLGDKDEEVDSWLLLSKDSDNNNNNNNNNDNDNNDNN       180
Kasalath         APLPAITIPATSVLAEAVVATATVLGGKDEEVDSWIILSKDSNNNNN-----------NN       169
Ginbouzu         APLPAITIPATSVLAEAVVATATVLGDKDEEVDSWLLLSKDSDNNNNNNNNNDNDNNDNN       180
Koshihikari      -PLPAITIPATSVLAEAVVATATVLGDKDEEVDSWLLLSKDSDNNNNNNNNNDNDNNDNN       168
                  *************************.********::*****:****           **

Akita63          NSNSSNNGMYFGEVDEYFDLVGYNSYYDNRIENNQDRQYGMHEQQEQQQQQQEMQKEFAE       240
Kasalath         NSNSSNNGMYFGEVDEYFDLVGYNSYYDNRIENNQDQQYGMHEQQEQQQQQQEMQKEFAE       229
Ginbouzu         NSNSSNNGMYFGEVDEYFDLVGYNSYYDNRIENNQDRQYGMHEQQEQQQQQQEMQKEFAE       240
Koshihikari      NSNSSNNGMYFGEVDEYFDLVGYNSYYDNRIENNQDRQYGMHEQQEQQQQQQEMQKEFAE       228
                 ************************************:***********************

Akita63          KEGSECVVMELWEQTRPPP-----------------------------------------       259
Kasalath         KEGSECVVPSQITMLSEQQHSGYGVVGADQAASMTAGVSAYTDSISNSISLINGGGYSTR       289
Ginbouzu         KEGSECVVPSQITMLSEQQHSGYGVVGADQAASMTAGVSAYTDSISNSISFSSMEAGIVP       300
Koshihikari      KEGSECVVPSQITMLSEQQHSGYGVVGADQAASMTAGVSAYTDSISNSISFSSMEAGIVP       288
                 ******** .

Akita63          ------------------------------------------------------------       259
Kasalath         QHG--D-RYAKFQHPD---TCWSNQSLLRSLA-----SDVPSLQLHGQGG----------       328
Ginbouzu         DSTVIDMPNSRILTPAGAINLFSGPSLQMSLHFSSMDREARVLRYREKKKARKFEKTIRY       360
Koshihikari      DSTVIDMPNSRILTPAGAINLFSGPSLQMSLHFSSMDREARVLRYREKKKARKFEKTIRY       348

Akita63          -----------------------------------------------       259
Kasalath         --------QGAQVQGEEE-----GQEV--------------------       342
Ginbouzu         ETRKAYAEARPRIKGRFAKRSDVQIEVDQMFSTAALSDGSYGTVPWF       407
Koshihikari      ETRKAYAEARPRIKGRFAKRSDVQIEVDQMFSTAALSDGSYGTVPWF       395
```

Supplementary Figure S4. (**b**) Alignment of Hd1 in Akita 63, Kasalath, Ginbouzu, and Koshihikari. Asterisks, dashes, periods, and colons indicate identical amino acid sequences, missing residues, semi-conservative substitutions, conservative substitutions, respectively.

```
Ginbouzu        MNYNFGGNVFDQEVGVGGEGGGGGEGSGCPWARPCDGCRAAPSVVYCRADAAYLCASCDA    60
Hoshinoyume     MNYNFGGNVFDQEVGVGGEGGGGGEGSGCPWARPCDGCRAAPSVVYCRADAAYLCASCDA    60
Hayamasari      MNYNFGGNVFDQEVGVGGEGGGGGEGSGCPWARPCDGCRAAPSVVYCRADAAYLCASCDA    60
                ************************************************************

Ginbouzu        RVHAANRVASRHERVRVCEACERAPAALACRADAAALCVACDVQVYSANPLARRHQRVPV    120
Hoshinoyume     RVHAANRVASRHERVRVCEACERAPAALACRADAAALCVACDVQVYSANPLARRHQRVPV    120
Hayamasari      RVHAANRVASRHERVRVCEACERAPAALACRADAAALCVACDVQVYSANPLARRHQRVPV    120
                ************************************************************

Ginbouzu        APLPAITIPATSVLAEAVVATATVLGDKDEEVDSWLLLSKDSDNNNNNNNNNDNDNNDNN    180
Hoshinoyume     APLPAITIPATSVLAEAVVATATVLGDKDEEVDSWLLLSKDSDNNNNNNNNNDNDNNDNN    180
Hayamasari      APLPAITIPATSVLAEAVVATATVLGDKDEEVDSWLLLSKDSDNNNNNNNNNDNDNNDNN    180
                ************************************************************

Ginbouzu        NSNSSNNGMYFGEVDEYFDLVGYNSYYDNRIENNQDRQYGMHEQQEQQQQQQEMQKEFAE    240
Hoshinoyume     NSNSSNNGMYFGEVDEYFDLVGYNSYYDNRIENNQDRQYGMHEQQEQQQQQQEMQKEFAE    240
Hayamasari      NSNSSNNGMYFGEVDEYFDLVGYNSYYDNRIENNQDRQYGMHEQQEQQQQQQEMQKEFAE    240
                ************************************************************

Ginbouzu        KEGSECVVPSQITMLSEQQHSGYGVVGADQAASMTAGVSAYTDSISNSISFSSMEAGIVP    300
Hoshinoyume     KEGSECVVPSQITMLSEQQHSGYGVVGADQAASMTAGVSAYTDSISNSISFSSMEAGIVP    300
Hayamasari      KEGSECVVPSQITMLSEQQHSGYGVVGADQAASMTAGVSAYTDSISNSISFSSMEAGIVP    300
                ************************************************************

Ginbouzu        DSTVIDMPNSRILTPAGAINLFSGPSLQMSLHFSSMDREARVLRYREKKKARKFEKTIRY    360
Hoshinoyume     DSTVIDMPNSRILTPAGAINLFSGPSLQMSLHFSSMDREARVLRYREKKKARKFEKTIRY    360
Hayamasari      DSTVIDMPNSRILTPAGAINLFSGPSLQMSLHFSSMDREARVLRYREKKKARKFEKTIRY    360
                ************************************************************

Ginbouzu        ETRKAYAEARPRIKGRFAKRSDVQIEVDQMFSTAALSDGSYGTVPWF     407
Hoshinoyume     ETRKAYAEARPRIKGRFAKRSDVQIEVDQMFSTAALSDGSYGTVPWF     407
Hayamasari      ETRKAYAEARPRIKGRFAKRSDVQIEVDQMFSTAALSDGSYGTVPWF     407
                ***********************************************
```

Supplementary Figure S4. (**c**) Alignment of Hd1 in Ginbouzu, Hoshinoyume, and Hayamasari. Asterisks indicate identical amino acid sequences.
